# *IL23R* and *ATG16L1* variants in Moroccan patients with inflammatory bowel disease

**DOI:** 10.1186/1756-0500-7-570

**Published:** 2014-08-26

**Authors:** Nadia Serbati, Nezha Senhaji, Brehima Diakite, Wafaa Badre, Sellama Nadifi

**Affiliations:** Laboratory of Medical Genetics– Medical school of Casablanca, Faculté de Médecine et de Pharmacie de Casablanca, 19, rue Tarik ibn ziad, Casablanca, Morocco; Department of Gastro-enterology, CHU IBN ROCHD, Casablanca, Morocco; Center Of Doctoral Sciences “In Health Sciences”, University Ain Chock Hassan Ii, Casablanca, Morocco

**Keywords:** IBD, *ATG16L1*, *IL23R*, Moroccan population

## Abstract

**Background:**

Inflammatory bowel diseases (IBD) are chronic diseases of the gastrointestinal tract. Although their pathogenesis is unclear, the combination of genetic predisposition and environmental components are believed to be the main cause of these diseases. Recently, many variants in interleukin 23 receptor (*IL23R*) and autophagy-related 16-like 1 (*ATG16L1*) genes have been associated with the disease. Our objective was to assess the frequency of *ATG16L1* (T300A) and *IL23R* (L310P) variants in Moroccan IBD (Crohn’s disease and Ulcerative Colitis) patients and to evaluate a possible effect of these variants on disease’s phenotype and clinical course.

**Methods:**

96 Moroccan IBD patients and 114 unrelated volunteers were genotyped for *ATG16L1* (T300A) and *IL23R* (L310P) variants by PCR-restriction fragment length polymorphism.

**Results:**

This is the first report on the prevalence of *ATG16L1* (T300A) and *IL23R* (L310P) variants in a Moroccan group. We found that *IL23R* (L310P) variant conferred a protective effect for crohn’s disease (CD) but not ulcerative colitis (UC) patients. The presence of *ATG16L1* (T300A) mutated alleles was associated with CD type but not with disease onset. In addition, the carriage of T300A variant alleles conferred a protective effect in UC.

**Conclusion:**

Our results showed that the prevalence of *ATG16L1* and *IL23R* variants was not significantly different between patients and controls. However a possible role of *ATG16L1* (T300A) on CD phenotype was suggested.

**Electronic supplementary material:**

The online version of this article (doi:10.1186/1756-0500-7-570) contains supplementary material, which is available to authorized users.

## Background

Inflammatory bowel disease (IBD) is a chronic and multifactorial disease of the gastrointestinal tract. It includes Crohn’s disease (CD), ulcerative colitis (UC) and undetermined colitis. Their etiologies remain complex and unclear involving an inadequately defined relationship between microbial insult, genetic predisposition and altered intestinal barrier permeability [1]. Several genetic studies have attempted to find out more about the molecular pathogenesis of CD and UC.

Genetic variations in genes related to innate and adaptive immunity have been implicated in IBD pathogenesis. Positive correlations were reported for Interleukin 23 receptor (*IL23R*) [2] and Autophagy related 16-like 1 (*ATG16L1*) [3, 4] genes.

IL-23 is a heterodimeric cytokine produced by activated macrophages and dendritic cells. It consists of two subunits, a p40 subunit, shared with the IL-12, and a specific IL-23 subunit called p19 [5, 6]. Studies have shown that IL-23 is involved in the initiation of the innate and adaptive immune activation that characterizes IBD. It binds a complex of IL-23R and IL-12Rβ subunits. IL-23R is predominantly expressed on activated/memory T cells, T-cell clones, natural killer’s (NK) cells and, at low levels, in monocytes, macrophages, and dendritic cell populations [7, 8]. Recent studies have shown the association of some single nucleotide polymorphisms (SNPs) in the IL-*23R* gene with chronic inflammatory diseases especially IBD (CD and UC). The variant L310P of *IL23R* gene (more frequent in controls) was reported to confer a strong protection against CD [2]. In Ulcerative colitis, the effect of this mutation seems to be insignificant [9]. In addition, Lin Z et al. suggested the role of IL23R (L310P) as a protective polymorphism in UC females [10].

Several studies have established an association between *ATG16L1* and IBD in various populations. The *ATG16L1* gene plays a key role in autophagy pathways. It encodes a protein widely expressed in intestinal epithelial cells, lymphocytes and macrophages and mediates resistance to intracellular pathogens such as bacteria and viral particles [11]. Hampe at al. reported an association between the T300A (c898G > A) polymorphism and Crohn’s disease [3]. Subsequent replication studies revealed divergent results.

No data were available on the frequency of the *ATG16L1* and *IL23R* variants in the Moroccan population. Hence, we aimed to examine the association between *IL23R* (L310P) and *ATG16L1* (T300A) polymorphisms and inflammatory bowel disease (Crohn’s disease and Ulcerative colitis) in a cohort of Moroccan patients.

## Methods

### Patients and controls

In this study, a group of 96 Moroccan unrelated IBD patients were recruited at the gastroenterology department of Averroes Hospital, Casablanca, Morocco. The control group included 114 unrelated Moroccan volunteers (blood donors) with no discernable symptoms suggestive of IBD. The diagnosis of CD or UC was based on established clinical, radiological, endoscopic, and histopathology criteria.

Demographic and clinical characteristics were obtained from the participants through a detailed questionnaire. CD phenotype was stratified by age at diagnosis, location and disease’s behaviour according to the Montreal classification [12].

For UC patients, anatomic location was subgrouped using the Paris classification as being ulcerative proctitis (E1), left-sided UC (E2), and extensive UC (E3) [13].

Differences in the frequency of disease characteristics such as age at diagnosis, gender, extra-intestinal manifestations, similar familial cases, and antecedents like appendectomy and smoking were also assessed. The study was approved by the medical school of Casablanca ethical committee. A written informed consent was obtained from all participants or their guardians. Both IBD patients and control group are originated from the different regions of Morocco and confirmed the Moroccan origin of their parents and grandparents.

### Genotyping methods

Genomic DNA was isolated from whole blood samples by salting-out method [6]. DNA amount and quality were measured by spectrophotometry. *IL23R* and *ATG16L1* variants genotyping was performed using polymerase chain reaction (PCR) restriction fragment length polymorphism analysis (RFLP) as described respectively by Lin et al. and Csöngei et al. [10, 14].

Reactions were performed in a final volume of 25 μl. PCR products were cleaved with Hph I (L310P) and Lwe I (T300A) (New England Biolabs Ipswich, UK) and electrophored on a 3% agarose gel in the presence of a molecular weight marker ladder 100 (New England Biolabs Ipswich, UK). After staining with ethidium bromide, Ultraviolet was used on a transilluminator for reading the gel.

### Statistical analysis

Statistical analysis was performed using MedCalc statistical software version 11.6. The Hardy-Weinberg equilibrium test was performed separately for patients and controls to measure the distribution of polymorphisms. The association between IBD (CD and UC) and *IL23R* (L301P) *ATG16L1* (T300A) genotypes was determined by Fisher's exact test (Odds Ratio with Confidence interval (CI) at 95%). The χ2 test or Fisher test was used to correlate the *IL23R* and *ATG16L1* polymorphisms and clinical parameters. The P value (<0.05) was considered statistically significant in all variables.

## Results

### Epidemiologic data

One hundred fourteen participants from the general population were genotyped for *ATG16L1* (T300A) and *IL23R* (L310P) along with 69 Crohn’s disease patients (25 women and 44 men) and 30 UC patients (14 women and 16 men). The average age of diagnosis was 24.17 ± 2.48 for CD patients and 35.37 ± 5 for UC patients. For control group, epidemiological and clinical data are shown in Additional file 1: Table S1.

### Genetic and clinical correlations

Statistical analysis of the distribution of SNPs studied showed that allele frequencies were conformed to Hardy–Weinberg expectations (=1.14, P = 0.57; =0.017, P = 0.99) (=0.03, P = 0.86; =0.017, P = 0.99) for T300A (*ATG16L1*) and L310P (*IL23R*) in CD patients and controls respectively.

Correlation between demographic and clinical characteristics according to *ATG16L1* and *IL23R* genotypes (Tables 1 and 2) revealed a positive association between CD Type and *ATG16L1* polymorphism (T300A) with P = 0.03 (Table 1). However, no genotype-phenotype correlation was noticed for the *IL23R* SNP.

**Table 1:** **Genotypic frequencies according to clinical parameters of the Moroccan CD patients investigated for the T300A polymorphism**

		ATG16L1 T300A			P value	Chi-square
	N	AA	AG	GG		Test
**Age of onset**	69				0.37	4.30
<17 years	10	-	8 (80.0)	2 (20.0)		
17-40	52	15 (28.8)	30 (57.7)	7 (13.5)		
>40 years	7	1 (14.3)	5 (71.4)	1 (14.3)		
**Sex**	69				0.57	1.14
Woman	25	4 (16.0)	17 (68.0)	4 (16.0)		
Man	44	12 (27.3)	26 (59.1)	6 (13.6)		
**Type**	69				0.03	13.7
Fistulizing	26	5 (19.2)	20 (76.9)	1 (3.8)		
Non fistulizing Non stenosing	24	7 (29.2)	10 (41.7)	7 (29.2)		
Stenosing	12	4 (33.3)	8 (66.7)	-		
Fistulizingstenosing	7	-	5 (71.4)	2 (28.6)		
**Localization**	69				0.36	17.4
L1	19	1 (5.3)	13 (68.4)	5 (26.3)		
L1 + P	2	1 (50.0)	1 (50.0)	-		
L2	10	4 (40.0)	4 (40.0)	2 (20.0)		
L2 + P	7	3 (42.9)	4 (57.1)	-		
L3	19	6 (31.6)	11 (57.9)	2 (10.5)		
L3 + P	1	-	1 (100.0)	-		
L4	2	-	1 (50.0)	1 (50.0)		
L4 + L2	4	1 (25.0)	3 (75.0)	-		
P	5	-	5 (100.0)	-		
**SFC**	69				0.09	4.8
Presence	4	-	2 (50.0)	2 (50.0)		
Absence	65	16 (24.6)	41 (63.1)	8 (12.3)		
**Smoking**	69				0.96	0.1
Presence	28	7 (25.0)	17 (60.7)	4 (14.3)		
Absence	41	9 (22.0)	26 (63.4)	6 (14.6)		
**Appendectomy**	69				0.22	3.1
Presence	9	2 (22.2)	4 (44.4)	3 (33.3)		
Absence	60	14 (23.3)	39 (65.0)	7 (11.7)		
**EIM**	69				0.97	0.1
Presence	39	9 (23.1)	24 (61.5)	6 (15.4)		
Absence	30	7 (23.3)	19 (63.3)	4 (13.3)		
**Surgery**	69				0.14	4.0
Presence	29	4 (13.8)	22 (75.9)	3 (10.3)		
Absence	40	12 (30.0)	21 (52.5)	7 (17.5)		

**(SFC:** Similar familial cases; **EIM:** Extra intestinal manifestations; **N:** Total number; **AA:** wild type ATG16L1 T300A, **AG:** ATG16L1 T300A heterozygous variant, **GG:** ATG16L1 T300A homozygous variant).

**Table 2:** **Genotypic frequencies according to clinical parameters of the Moroccan CD patients investigated for the L310P polymorphism**

		IL23R L310P			P value	Chi-square
	N	CC	CT	TT		Test
**Age of onset**	69				0.16	3.7
<17 years	10	6 (60.0)	4 (40.0)			
17-40	52	40 (76.9)	12 (23.1)			
>40 years	7	7 (100.0)				
**Sex**	69				0.86	0.0
Woman	25	19 (76.0)	6 (24.0)			
Man	44	34 (77.3)	10 (22.7)			
**Type**	69				0.11	6.1
Fistulizing	26	20 (76.9)	6 (23.1)			
Non fistulizing Non stenosing	24	21 (87.5)	3 (12.5)			
Stenosing	12	9 (75.0)	3 (25.0)			
Fistulizingstenosing	7	3 (42.9)	4 (57.1)			
**Localization**	69				0.29	9.6
L1	19	16 (84.2)	3 (15.8)			
L1 + P	2	2 (100.0)	-			
L2	10	6 (60.0)	4 (40.0)			
L2 + P	7	7 (100.0)	-			
L3	19	13 (68.4)	6 (31.6)			
L3 + P	1	-	1 (100.0)			
L4	2	2	-			
L4 + L2	4	3 (75.0)	1 (25.0)			
P	5	4 (80.0)	1 (20.0)			
**SFC**	69				0.60	0.3
Presence	4	4 (100.0)	-			
Absence	65	49 (75.4)	16 (24.6)			
**Smoking**	69				1.0	0.0
Presence	28	22 (78.6)	6 (21.4)			
Absence	41	31 (75.6)	10 (24.4)			
**Appendectomy**	69				0.73	0.12
Presence	9	6 (66.7)	3 (33.3)			
Absence	60	47 (78.3)	13 (21.7)			
**EIM**	69				0.14	2.14
Presence	39	33 (84.6)	6 (15.4)			
Absence	30	20 (66.7)	10 (33.3)			
**Surgery**	69				0.30	1.1
Presence	29	20 (69.0)	9 (31.0)			
Absence	40	33 (82.5)	7 (17.5)			

**(SFC:** Similar familial cases; **EIM:** Extra intestinal manifestations; **N:** Total number; **CC:** wild type IL23R L310P, **CT:** IL23R L310P heterozygous variant, **TT:** IL23R L310P homozygous variant).

Case–control studies were carried out for the selected polymorphisms. The genotypic and allelic frequencies for the T300A and L310P polymorphisms are presented in Tables 3 and 4 respectively.

**Table 3:** **Genotypic and allelic frequencies for the ATG16L1 of CD patients and controls**

Genotype allele	Case (%) N = 69	Controls (%) N = 115	OR (0.95 CI)	P value
**AA**	16 (23.2)	30 (26.1)	1.0	
**AG**	43 (62.3)	76 (66.1)	1.06 (0.52-2.16)	0.87
**GG**	10 (14.5)	9 (7.8)	2.08 (0.70-6.17)	0.19
**A**	75 (54.3)	136 (59.1)	1.0	
**G**	63 (45.7)	94 (40.9)	1.22 (0.79-1.86)	0.36

**(AA:** wild type ATG16L1 T300A, **AG:** ATG16L1 T300A heterozygous variant, **GG:** ATG16L1 T300A homozygous variant; **N:** Total number; **OR:** odd ratio; **CI** confidence interval; **P:** (P < 0.05)).

**Table 4:** **Genotypic and allelic frequencies for the IL23R of CD patients and controls**

Genotype allele	Case (%) N = 69	Controls (%) N = 115	OR (95% CI)	P value
**CC**	53 (76.8)	98 (85.2)	1.0	
**CT**	8 (23.2)	14 (12.2)	1.06 (0.41-2.68)	0.91
**TT**	0.0	3 (2.6)	0.26 (0.01-5.19)	0.38
**C**	114 (82.6)	210 (91.3)	1.0	
**T**	8 (17.4)	20 (8.7)	0.74 (0.31-1.73)	0.48

**CC:** wild type IL23R L310P, **CT:** IL23R L310P heterozygous variant, **TT:** IL23R L310P homozygous variant; **N:** Total number; **OR:** odd ratio; **CI** confidence interval; **P:** (P < 0.05).

The non-synonymous polymorphism, rs2241880 (Thr300Ala), located on the *ATG16L1* gene, showed no significantly increased risk of CD among individuals carrying GG genotype or G allele with the respective odds ratio 2.08 (CI: 0.70-6.17, P = 0.19); 1.22 (CI: 0.79-1.86, P = 0.36) (Table 5). In addition, individuals carrying the mutated allele are not protected from the disease. In contrast to the L310P polymorphism in *IL23R* gene, which confers protection to individuals with the TT genotype and T allele against the development of Crohn’s disease, with respective odds ratio 0.26 (CI: 0.01-5.19, P = 0.38) and 0.74 (CI: 0.31-1.73, P = 0.48) (Table 4).

**Table 5:** **Genotypic frequencies according to clinical parameters of the Moroccan UC patients investigated for the T300Apolymorphism**

Clinical parameters	N	ATG16L1T300A			P value	Chi-deux test
		AA	AG	GG		
**Age of onset**	30				0.12	4.32
<17 years	-					
17-40	21	10 (47.6)	8 (38.1)	3 (14.3)		
>40 years	9	1 (11.1)	7 (77.8)	1 (11.1)		
**Sex**	30				0.41	1.76
Woman	14	4 (28.6)	7 (50.0)	3 (21.4)		
Man	16	7 (43.8)	8 (50.0)	1 (6.3)		
**Localization**	30				0.71	3.73
Leftcolitis	11	5 (45.5)	5 (45.5)	1 (9.1)		
Extensive colitis	2	-	2 (100.0)	-		
Pancolitis	13	4 (30.8)	7 (53.8)	2 (15.4)		
Proctitis	4	2 (50.0)	1 (25.0)	1 (25.0)		
**SFC**	30				0.41	1.79
Presence	1	1 (100.0)	-	-		
Absence	29	10 (34.5)	15 (51.7)	4 (13.8)		
**Smoking**	30				0.19	3.36
Presence	8	5 (62.5)	2 (25.0)	1 (12.5)		
Absence	22	6 (27. 3)	13 (59.1)	3 (13.6)		
**Appendectomy**	30				0.05	6.20
Presence	-	-	-	-		
Absence	30	11 (36.7)	15 (50.0)	4 (13.3)		
**EIM**	30				0.50	1.38
Presence	17	7 (41.2)	7 (41.2)	3 (17.6)		
Absence	13	4 (30.8)	8 (61.5)	1 (7.7)		
**Surgery**	30				0.22	3.04
Presence	4	3 (75.0)	1 (25.0)	-		
Absence	26	8 (30.8)	14 (53.8)	4 (15.4)		

**(SFC:** Similar familial cases; **EIM:** Extra intestinal manifestations; **N:** Total number; **AA:** wild type ATG16L1 T300A, **AG:** ATG16L1 T300A heterozygous variant, **GG:** ATG16L1 T300A homozygous variant).

Additionally, our study assessed the association of *ATG16L1* (T300A) and *IL23R* (L310P) polymorphisms with UC. Analysis of distribution of the two polymorphisms showed that allele frequencies were in Hardy-Weinberg equilibrium (=1.76, P = 0.41 and =0.017, P = 0.99) for *ATG16L1* and *IL23R* (=2.9, P = 0.23; =0.017, P = 0.99).

For both polymorphisms, no genotype-phenotype correlation was observed in UC (Tables 5 and 6).

**Table 6:** **Genotypic frequencies according to clinical parameters of the Moroccan UC patients investigated for the L310P polymorphism**

Clinical parameters	N	IL23L310P			P value	Chi-deux test
		CC	CT	TT		
**Age of onset**	30				0.31	2.34
<17 years	-	-	-	-		
17-40	21	16 (76.2)	4 (19.0)	1 (4.8)		
>40 years	9	5 (55.6)	4 (44.4)	-		
**Sex**	30				0.30	2.93
Woman	14	11 (78.6)	2 (14.3)	1 (7.1)		
Man	16	10 (62.5)	6 (37.5)	-		
**Localization**	30				0.43	5.95
Leftcolitis	11	6 (54.5)	5 (45.5)	-		
Extensive colitis	2	1 (50.0)	1 (50.0)	-		
Pancolitis	13	10 (76.9)	2 (15.4)	1 (7.7)		
Proctitis	4	4 (100.0)	-	-		
**SFC**	30				0.80	0.44
Presence	1	1 (100.0)	-	-		
Absence	29	20 (69.0)	8 (27.6)	1 (3.4)		
**Smoking**	30				0.81	0.41
Presence	8	6 (75.0)	2 (25.0)	-		
Absence	22	15 (68.2)	6 (27.3)	1 (4.5)		
**Appendectomy**	30				<0.0001	20.60
Presence	-	-	-	-		
Absence	30	21 (70.0)	8 (26.7)	1 (3.3)		
**EIM**	30				0.60	1.03
Presence	17	11 (64.7)	5 (29.4)	1 (5.9)		
Absence	13	10 (76.9)	3 (23.1)	-		
**Surgery**	30				0.37	1.98
Presence	26	17 (65.4)	8 (30.8)	1 (3.8)		
Absence	4	4 (100.0)	-	-		

**(SFC:** Similar familial cases; **EIM:** Extra intestinal manifestations; **N:** Total number; **CC:** wild type IL23R L310P, **CT:** IL23R L310P heterozygous variant, **TT:** IL23R L310P homozygous variant).

The genotypic and allelic frequencies did not significantly differ between UC patients and healthy controls for the two polymorphisms (Tables 7 and 8).

**Table 7:** **Genotypic and allelic frequencies for the ATG16L1 of UC patients and controls**

Genotype allele	Case (%) N = 30	Controls (%) N = 115	OR (0.95 CI)	P value
**AA**	11 (36.7.)	30 (26.1)	1.0	
**AG**	15 (50.0)	76 (66.1)	0.54 (0.22-1.30)	0.17
**GG**	4 (13.3)	9 (7.8)	1.21 (0.31-4.75)	0.78
**A**	37 (61.7)	136 (59.1)	1.0	
**G**	23 (38.3)	94 (40.9)	0.90 (0.50-1.61)	0.72

**(AA:** wild type ATG16L1 T300A, **AG:** ATG16L1 T300A heterozygous variant, **GG:** ATG16L1 T300A homozygous variant; **N:** Total number; **OR:** odd ratio; **CI** confidence interval; **P:** (P < 0.05)).

**Table 8:** **Genotypic and allelic frequencies for the IL23R of UC patients and controls**

Genotype allele	Case (%) N = 30	Controls (%) N = 115	OR (0.95 CI)	P value
**CC**	21 (70.0)	98 (85.2)	1.0	
**CT**	8 (26.7)	14 (12.2)	2.67 (0.99-7.16)	0.05
**TT**	1 (3.3)	3 (2.6)	1.56 (0.15-15.70)	0.71
**C**	50 (83.3)	210 (91.3)	1.0	
**T**	10 (16.7)	20 (8.7)	2.10 (0.92-4.77)	0.08

**CC:** wild type IL23R L310P, **CT:** IL23R L310P heterozygous variant, **TT:** IL23R L310P homozygous variant; **N:** Total number; **OR:** odd ratio; **CI** confidence interval; **P:** (P < 0.05).

Carriers of mutated allele in *ATG16L1* gene have a protective effect for UC, with an odds ratio of 0.90 (CI: 0.50-1.61, P = 0.72) (Table 7). While carriers of mutated allele in *IL23R* gene are not protected from UC, with an OR of 2.10 (CI: 0.92-4.77, P = 0.08) (Table 8).

## Discussion

### ATG16L1polymorphism

The association of genes within the autophagy pathway with IBD was observed in several studies. One of the prime candidate genes discovered was the *ATG16L1* gene, ATG16L1 is a protein expressed in the colon, leukocytes, intestinal epithelial cells, small intestine, and spleen [15]. A mutation on the gene encoding this protein, located on chromosome 2, has been associated with the onset of ileal CD [16]. It has been shown that ATG16L is a key molecule in elucidating the genetic aspects of CD. The findings of associations with variants in *ATG16L1* and IBD have prompted further research on understanding the role of the autophagy pathway in disease pathogenesis.

During a genome-wide survey of 19 779 non-synonymous single nucleotide polymorphisms, the (Thr300Ala) variant, located at the N terminus of the WD-repeat domain in ATG16L1, was found to be highly associated with CD by using a haplotype and regression analysis [3]. Subsequent to the initial genome wide association study, many studies have consistently identified associations between the *ATG16L1* (Thr300Ala) variant and CD [17, 18]. This finding has been widely replicated in different populations [19–32].

In the present study, we examined the association of *ATG16L1* (T300A) genetic variant with CD and UC in Moroccan patients and controls. Upon association analysis, we were not able to establish a significant effect on CD risk in Moroccan IBD cohort. Our result was in concordance with the lack of association reported in a replication study performed in Japan [33]. In addition, Van Limbergen et al. [34] observed that the *ATG16L1* variant is associated with susceptibility to adult CD, but not with early-onset disease in a Scottish cohort.

Regarding UC, a protective effect of this polymorphism was identified. At present, it can only be speculated how *ATG16L1* T300A variant may confers risk or protection from infection, depending on the nature of the pathogen and the typical duration of infection. The cellular expression of *ATG16L1* facilitates bacterial invasion, however the IBD-associated *ATG16L1* T300A variant may be protective against bacterial infection.

Messer et al. demonstrated that Intestinal epithelial cells somatically targeted to express the *ATG16L1* T300A variant show protection against invasion by Salmonella [23].

### IL23R polymorphism

The *IL23R* gene is another potential candidate gene for CD risk [2, 35]. IL-23R interacts with IL-23, which is a cytokine that orchestrates intestinal inflammation via multiple pathways. It regulates the activity of immune cells and plays an important role in the inflammatory response against infection by bacteria and viruses [36]. The IL-23-IL17 axis is a key pathogenic mechanism that mediates the development and progress of inflammation by Th-17 cells. The role of the IL23-IL17 axis in IBD was supported in human patients and animal models of colitis [37–39]. Similarly, several studies have pinpointed IL23 receptor as a key pathway in the pathogenesis of inflammatory bowel disease. It was confirmed by the genetic association of several SNPs throughout the *IL23R* gene with CD and UC [21, 22, 24–27, 30, 32, 40–43].

It was hypothesized that *IL23R* gene variants have a differential effect on Th17 cells with increased Th17 cytokine secretion in patients with CD-associated *IL23R* variants and decreased cytokine secretion in patients with CD-protective *IL23R* variants [44].

In the present study, carriage of the variant allele was associated with a protective effect for CD patients, similarly to previously reported studies [45, 46]. We further analyzed whether the risk factor in the *IL23R* gene was also shared by UC patients and did not detect a significant association. Our subgroup analyses are likely underpowered for revealing a genotype– phenotype relationship. This result confirms previous studies on Italian [47] and North American populations [42].

## Conclusion

In summary, the present study seems to indicate that *ATG16L1* plays an important role in CD behaviour and confers protection for UC. In addition, *IL23R* gene showed a protective effect for individuals with the TT genotype and T allele against the development of Crohn’s disease.

Therefore, our results could reinforce the notion of a different relevance of *ATG16L1* and *IL23R* in the pathogenesis of IBD in patients of different ethnic origin, with a limited role in the Moroccan population. Due to small sample size, an association cannot be ruled out. Further studies in larger groups would be required to confirm these findings.

## Electronic supplementary material

Additional file 1: Table S1: Clinical and epidemiological parameters of control group. (DOCX 11 KB)
