# Supplementary material for: IL23R and ATG16L1 variants in Moroccan patients with inflammatory bowel disease
Source: BMC Res Notes. 2014 Aug 26;7:570. doi: 10.1186/1756-0500-7-570 (PMC4162942; doi:10.1186/1756-0500-7-570)
Supplement: Supplementary file 1 — Additional file 1: Table S1: Clinical and epidemiological parameters of control group. (DOCX 11 KB) [file 13104_2014_3122_MOESM1_ESM.docx]

Additional Table S1: Clinical and epidemiological parameters of control group

| **Age** | **< 17 years old** | 16 |
| --- | --- | --- |
|  | **17-40 years old** | 68 |
|  | **> 40 ans years old** | 30 |
| **Sex** | **Men** | 50 |
|  | **Women** | 64 |
| **Ethnic Origin** | **Arab** | 66 |
|  | **Berber** | 48 |
| **Clinical signs of IBD/ Familial history of IBD** | | None |
